# Fecal Microbiota Transplantation, Commensal *Escherichia coli* and *Lactobacillus johnsonii* Strains Differentially Restore Intestinal and Systemic Adaptive Immune Cell Populations Following Broad-spectrum Antibiotic Treatment

**DOI:** 10.3389/fmicb.2017.02430

**Published:** 2017-12-11

**Authors:** Ira Ekmekciu, Eliane von Klitzing, Christian Neumann, Petra Bacher, Alexander Scheffold, Stefan Bereswill, Markus M. Heimesaat

**Affiliations:** ^1^Intestinal Microbiology Research Group, Institute of Microbiology, Charité – Universitätsmedizin Berlin, corporate member of Freie Universität Berlin, Humboldt-Universität zu Berlin, and Berlin Institute of Health, Berlin, Germany; ^2^Department of Cellular Immunology, Clinic for Rheumatology and Clinical Immunology, Charité – Universitätsmedizin Berlin, corporate member of Freie Universität Berlin, Humboldt-Universität zu Berlin, and Berlin Institute of Health, Berlin, Germany; ^3^German Rheumatism Research Center, Leibniz Association, Berlin, Germany

**Keywords:** commensal intestinal microbiota, *Escherichia coli*, *Lactobacillus johnsonii*, fecal microbiota transplantation, secondary abiotic (gnotobiotic) mice, intestinal mucosal and peripheral and central immunity, immune-modulating effects, immune restoration

## Abstract

The essential role of the intestinal microbiota in the well-functioning of host immunity necessitates the investigation of species-specific impacts on this interplay. Aim of this study was to examine the ability of defined Gram-positive and Gram-negative intestinal commensal bacterial species, namely *Escherichia coli* and *Lactobacillus johnsonii*, respectively, to restore immune functions in mice that were immunosuppressed by antibiotics-induced microbiota depletion. Conventional mice were subjected to broad-spectrum antibiotic treatment for 8 weeks and perorally reassociated with *E. coli*, *L. johnsonii* or with a complex murine microbiota by fecal microbiota transplantation (FMT). Analyses at days (d) 7 and 28 revealed that immune cell populations in the small and large intestines, mesenteric lymph nodes and spleens of mice were decreased after antibiotic treatment but were completely or at least partially restored upon FMT or by recolonization with the respective bacterial species. Remarkably, *L. johnsonii* recolonization resulted in the highest CD4+ and CD8+ cell numbers in the small intestine and spleen, whereas neither of the commensal species could stably restore those cell populations in the colon until d28. Meanwhile less efficient than FMT, both species increased the frequencies of regulatory T cells and activated dendritic cells and completely restored intestinal memory/effector T cell populations at d28. Furthermore, recolonization with either single species maintained pro- and anti-inflammatory immune functions in parallel. However, FMT could most effectively recover the decreased frequencies of cytokine producing CD4+ lymphocytes in mucosal and systemic compartments. *E. coli* recolonization increased the production of cytokines such as TNF, IFN-γ, IL-17, and IL-22, particularly in the small intestine. Conversely, only *L. johnsonii* recolonization maintained colonic IL-10 production. In summary, FMT appears to be most efficient in the restoration of antibiotics-induced collateral damages to the immune system. However, defined intestinal commensals such as *E. coli* and *L. johnsonii* have the potential to restore individual functions of intestinal and systemic immunity. In conclusion, our data provide novel insights into the distinct role of individual commensal bacteria in maintaining immune functions during/following dysbiosis induced by antibiotic therapy thereby shaping host immunity and might thus open novel therapeutical avenues in conditions of perturbed microbiota composition.

## Introduction

Approximately 10^13^ microorganisms, collectively known as microbiota, reside in the human gastrointestinal tract (Sender et al., 2016) and have increasingly received well-deserved attention regarding their deep impact on the physiology and well-being of the mammalian host. The microbiota composition is characterized by vast inter-individual variations and is furthermore influenced by numerous factors, including genetics (Org et al., 2015), mode of delivery (Biasucci et al., 2010), age (Palmer et al., 2007), diet (Ericsson and Franklin, 2015), hospitalization (Penders et al., 2006), exposure to pathogens and/or antibiotics (Buffie et al., 2012; Carding et al., 2015). It has been shown that bacteria belonging to the phyla *Firmicutes*, *Bacteroidetes*, *Proteobacteria*, *Actinobacteria*, and *Fusobacteria* comprise the vast majority of the mammalian intestinal microbiota (Eckburg et al., 2005; Palmer et al., 2007; Hill et al., 2012) and both bacterial density and diversity increase from the proximal to the distal gut (Sommer and Backhed, 2013). *Firmicutes* are Gram-positive bacteria, which include the large class of *Clostridia* and the lactic acid bacteria (Zhang et al., 2015), while *Escherichia coli* (Gram-negative, rod-shaped bacteria belonging to the family *Enterobacteriaceae* and phylum *Proteobacteria*) represent the predominant facultative anaerobe member of the mammalian gastrointestinal tract (Finegold et al., 1983; Tenaillon et al., 2010). The small intestinal microbiota is dominated by the families *Lactobacillaceae* and *Enterobacteriaceae*, whereas species from the families *Bacteroidaceae*, *Prevotellaceae*, *Rikenellaceae*, *Lachnospiraceae*, and *Ruminococcaceae* can be detected in the colon (Donaldson et al., 2016). While the microbiota is of paramount importance for numerous metabolic processes, including vitamin synthesis (LeBlanc et al., 2013) and digestion of dietary compounds (Backhed et al., 2005), compelling evidence points toward its impact on the maturation, development and function of the innate and adaptive immune system of the host (Macpherson and Harris, 2004; Sommer and Backhed, 2013).

Lactic acid bacteria exert important functions in the modulation of immune responses and have successfully been used as probiotics in inflammatory conditions of mice and men. For instance, *Lactobacillus rhamnosus* GG has been shown to exert preventive and therapeutic effects in atopic eczema and dermatitis (Isolauri et al., 2000; Kalliomaki et al., 2001, 2003; Viljanen et al., 2005). Moreover, treating IL-10 deficient mice with *L. plantarum* attenuated the severity of colonic inflammation by reducing mucosal IL-12p40 and IFN-γ levels (Schultz et al., 2002). In our own previous work, we could further demonstrate that a single commensal *L. johnsonii* strain was able to attenuate both intestinal mucosal and systemic pro-inflammatory immune responses upon murine infection with the enteropathogen *Campylobacter jejuni* (Bereswill et al., 2017). Kwon et al. (2010) have proposed that the observed beneficial anti-inflammatory properties may be elicited due to induction of regulatory DCs and regulatory T cells (Treg).

Gram-negative commensals, such as members of the family *Enterobacteriaceae*, tend to be neglected, but may, nevertheless, be potent immune-modulators (Zeuthen et al., 2006). This is best corroborated by the probiotic strain *E. coli* Nissle 1917, which has conclusively been shown efficient in the treatment of ulcerative colitis and is considered as an effective alternative to the standard maintenance therapy, i.e., mesalazine (Kruis et al., 2004; Henker et al., 2008; Schultz, 2008).

Importantly, Hessle et al. (2000, 2005) and Skovbjerg et al. (2010) have shown the differing impact of Gram-positive and Gram-negative bacteria on the modulation of cytokine production patterns, given that *in vitro* co-culturing of human PBMC with Gram-positive bacteria leads to elevated levels of IL-12, IL-1β, IFN-γ, and TNF, while Gram-negative bacteria rather induce IL-6, IL-8, and IL-10. In contrast, from monocytes derived human DC have been shown to produce comparable levels of IL-12 and TNF in response to commensal Gram-positive and Gram-negative bacteria (Karlsson et al., 2004; Zeuthen et al., 2006).

Murine studies have further underlined the impact of individual bacterial species on distinct immune cell populations. For instance, segmented filamentous bacteria (SFB) have been identified to induce the expansion of IL-17 producing Th17 cells (Ivanov et al., 2008), while *Clostridium* species of clusters IV and XIVa promoted accumulation of Treg in the colonic lamina propria (LP) of mice (Atarashi et al., 2011). The differentiation of CD4+ T cells into Treg locally in the LP as well as in the circulation is also supported by TLR-2 mediated sensing of the Gram-negative bacterium *Bacteroides fragilis* (Round et al., 2011). Moreover, treatment of IL-10^-/-^ mice with *L. plantarum* has been shown to attenuate the severity of colonic inflammation by reducing mucosal IL-12p40 and IFN-γ levels (Schultz et al., 2002). However, elucidating the differential impact of Gram-positive and Gram-negative commensals on the immune cell homeostasis in *in vivo* models, and identifying the species specific mechanisms of immunomodulation remains difficult and challenging, yet of utmost interest.

In our previous works, we have shown that normally developed mice rendered void of intestinal microbiota through a quintuple antibiotic therapy (i.e., secondary abiotic mice, ABx mice), display numerous changes of intestinal mucosal and systemic immune cell subsets, which can, however, almost completely be restored through reintroduction of intestinal antigens via fecal microbiota transplantation (FMT) (Ekmekciu et al., 2017a,b). Furthermore we were able to demonstrate that recolonization of ABx mice with VSL#3, a probiotic mixture consisting of eight bacterial species (namely *Streptococcus thermophilus*, *Bifidobacterium breve*, *B. longum*, *B. infantis*, *Lactobacillus acidophilus*, *L. plantarum*, *L. paracasei*, and *L. delbrueckii* subsp. *bulgaricus*), elicits IL-10 production by lymphocytes in the small and large intestinal LP, MLN and spleen, but does not influence the production of pro-inflammatory cytokines (Ekmekciu et al., 2017a,b). The impact of probiotics on cytokine production has been extensively investigated, and it has been proposed, that some strains might promote mainly IL-12 production, thus inducing Th1 type immune responses, which are known to protect from pathogens. Other strains, however, more effectively impacted the production of the anti-inflammatory IL-10, thereby limiting excessive immune responses as observed in autoimmune and allergic diseases, for instance (Fujiwara et al., 2004; Foligne et al., 2007; Shida et al., 2011).

Niess et al. (2008) have previously further addressed the role of commensal organisms in the recruitment of pro-inflammatory cytokine producing CD4+ cells in the colonic lamina propria and proposed that these cell types might contribute to the immunopathogenesis of colitis. However, a fine-tuned balance between pro- and anti-inflammatory immune responses at mucosal and systemic sites is of utmost importance for the vertebrate host. For instance, a reduction of the IL-17 producing Th17 cell compartment playing a pivotal role in protection against bacterial and fungal pathogens (Aujla et al., 2007) may explain the increased susceptibility of microbiota depleted mice to pathogens (Croswell et al., 2009; Stiemsma et al., 2014).

In the present study, we investigated the role of two important representatives of Gram-negative (namely *E. coli*) and Gram-positive (namely *L. johnsonii*) commensals, both derived from the gut microbiota of healthy mice, in restoring numbers of distinct immune cell subsets following antibiotic treatment as compared to FMT in C57Bl/6j ABx mice. To address this, we analyzed the immune responses exerted by lymphocytes within the small and large intestinal LP, MLN and spleen of microbiota-depleted mice upon reassociation with *E. coli* or *L. johnsonii* and upon FMT at day (d) 7 and d28 post recolonization. We furthermore surveyed the cytokine production patterns assessing TNF, IFN-γ, IL-17, IL-22, and IL-10 secretion by CD4+ lymphocytes in respective compartments.

## Materials and Methods

### Ethics Statement

All animal experiments were conducted according to the European Guideline for animal welfare (2010/63/EU) with approval from the commission for animal experiments headed by the “Landesamt für Gesundheit und Soziales” (LaGeSo, Berlin, Germany, registration numbers G0097/12 and G0184/12). Animal welfare was examined twice daily by assessment of clinical conditions including weight loss.

### Generation of Secondary Abiotic (Gnotobiotic) Mice

All animals were bred, raised and housed in the facilities of the “Forschungseinrichtungen für Experimentelle Medizin” (FEM, Charité – University Medicine Berlin, Germany) under specific pathogen-free (SPF) conditions. Secondary abiotic mice were generated through quintuple antibiotic treatment for 8 weeks via the drinking water as previously described (Heimesaat et al., 2006; Bereswill et al., 2011; Fiebiger et al., 2016; Ekmekciu et al., 2017a,b).

### Bacterial Recolonization

Three days prior bacterial recolonization experiments, the antibiotic cocktail was withdrawn and replaced by sterile drinking water (*ad libitum*). Successful depletion of the intestinal microbiota was confirmed and FMT performed as described previously (Ekmekciu et al., 2017a,b). Furthermore, secondary abiotic mice were recolonized with 10^9^ CFU of either *E. coli* or *L. johnsonii* in 0.3 ml PBS (Gibco Life Technologies, Paisley, United Kingdom) by gavage on day 0. The applied *E. coli* strain constitutes a commensal isolate derived from a naive conventionally colonized C57BL/6j wildtype mouse and did not express known virulence factors such as stx 1 and 2, hlyA, cspA, catA, katA, and astA as confirmed in a reference laboratory (Heimesaat et al., 2007; Haag et al., 2012). The *L. johnsonii* strain had initially been isolated from the feces of a healthy female 3 months old C57BL/6j wildtype mouse as previously described (Bereswill et al., 2017). For FMT fresh murine fecal samples were collected from 10 age and sex matched SPF control mice, pooled, dissolved in 10 ml sterile PBS and the supernatant perorally applied by gavage (in 0.3 ml PBS) in order to reconstitute secondary abiotic (i.e., gnotobiotic) mice with a complex intestinal microbiota as shown previously (Heimesaat et al., 2006; Ekmekciu et al., 2017a,b).

### Sampling Procedures

Mice were sacrificed by isoflurane treatment (Abbott, Greifswald, Germany) at d7 or d28 following bacterial recolonization. Luminal large intestinal samples as well as *ex vivo* biopsies from spleen, MLN, ileum, and colon were taken under sterile conditions. Intestinal samples were collected in parallel for microbiological and immunological analyses.

### Quantitative Analysis of Bacterial Colonization

Total intestinal loads of *E. coli* and *L. johnsonii* were quantitated in fecal and colonic samples over time post recolonization or upon necropsy. Respective samples were dissolved in PBS and serial dilutions streaked onto appropriate solid culture media: *E. coli* was detected on Columbia agar supplemented with 5% sheep blood and MacConkey agar (Oxoid, Wesel, Germany) following aerobic incubation at 37°C for 48 h. *L. johnsonii* loads were determined on Columbia agar supplemented with 5% sheep blood, Columbia-CNA agar supplemented with colistin and nalidixic acid and MRS agar (all from Oxoid) in parallel and incubated under microaerobic and obligate anaerobic conditions (in jars using CampyGen and AnaeroGen gas packs, respectively; both from Oxoid) at 37°C for at least 2 days. Bacterial species were identified according to their typical morphological appearances and biochemical properties. The detection limit of viable bacteria was ≈100 CFU/g.

### Lymphocyte Isolation from Spleen and Mesenteric Lymph Nodes

Single cell suspensions were generated from spleens and MLN, and erythrocytes were removed from splenic samples by 1.66% ammonium chloride. All samples were resuspended in volumes of 5 ml (spleen) and 2 ml (MLN) PBS/0.5% BSA (Sigma–Aldrich, St. Louis, MO, United States) and subjected to further processing (Cording et al., 2013).

### Lamina Propria Lymphocyte Isolation

Lamina propria lymphocyte isolation followed a standard protocol with minor modifications as described previously (Sheridan and Lefrancois, 2012; Ekmekciu et al., 2017b). Briefly, the intestines were cut into 0.5 cm pieces and incubated twice in 25 ml 1 mM dithioerythritol (DTE; Carl Roth) in Hanks balanced salt solution (HBSS; Gibco) for 20 min at 37°C at 220 rpm. Afterward the intestines were introduced to 1.3 mM ethylenediaminetetraacetic acid (EDTA; life technologies, Eugene, OR, United States) solution in HBSS and were shaken again twice herein for 30 min at 37°C at 220 rpm. After each incubation, the epithelial cell layer containing the intraepithelial lymphocytes was removed by intensive vortexing and passing through a 70 μm cell strainer, and new solution (DTE or EDTA) was added. After the second EDTA incubation the cells were washed with RPMI 1640 (Gibco) containing 5% fetal calf serum (FCS; Biochrom, Berlin, Germany) and were subsequently placed in 35 ml digestion solution containing 0.5 mg/ml collagenase A (Roche, Mannheim, Germany), 0.5 mg/ml DNAse I (Roche), 10% FCS, 1 mM of each CaCl_2_ and MgCl_2_ (both Carl Roth) in RPMI 1640 (Gibco). Digestion was performed through incubation for 45 min at 37°C and 220 rpm. After incubation the digested tissues were washed in RPMI supplemented with 5% FCS and centrifuged for 6 min at 4°C and 350 × *g*. The pellets were resuspended in 5 ml 44% Percoll (GE Healthcare, Uppsala, Sweden) and overlaid on 5 ml 67% Percoll in a 15 ml Falcon tube. Percoll gradient separation was performed by centrifugation at 600 × *g* for 20 min at room temperature. LPL were collected from the interphase, washed once and suspended in 1 ml of PBS/0.5% BSA.

### Surface and Intracellular Stainings and Flow Cytometry

Surface staining was performed using a mix of the following antibodies: FITC-anti-CD4 (Clone RM4-5; 1:200), PerCP-anti-CD8 (Clone 53-6.7; 1:100), PacBlue-anti-B220 (Clone RA3-6B2, 1:200), APC-Cy7-anti-CD25 (Clone PC61, 1:200), PE-anti-CD44 (Clone IM7, 1:200), APC-anti-CD86 (Clone B7-2, 1:200) (all from BD Biosciences, San Jose, CA, United States).

For intracellular staining cells from spleen, MLN and intestinal LP were restimulated for 5 h with 10 ng/ml phorbol myristate acetate (PMA) and 1 μg/ml ionomycin, in a tissue culture incubator at 37°C (both Sigma–Aldrich). The 10 μg/ml brefeldin A (Sigma–Aldrich) were added to the cell suspensions after 1 h of polyclonal restimulation. Then cells were treated with LIVE/DEAD Fixable Aqua Dead Cell Stain kit (life technologies) and hereafter fixed with 2% paraformaldehyde (PFA; Sigma–Aldrich) for 20 min at room temperature. Cells were stained in 0.5% saponin (Sigma–Aldrich) using a mix of the following antibodies: PacBlue-Anti-CD4 (Clone RM4-5; 1:400), PE-Cy7-anti-IFN-γ (Clone XMG 1.2; 1:400), APC-Cy7-anti-TNF-α (Clone MP6-XT22; 1:400) (all three from BD Biosciences), FITC-anti-IL17A (Clone TC11-18H10.1; 1:200, BioLegend, San Diego, CA, United States), PE-anti-IL10 (Clone JESS-16E3; 1:100), APC-anti-IL22 (Clone IL22JOP; 1:100) (both from eBioscience). After gating for lymphocytes and excluding doublets only living cells were included in further analyses. CD4 and CD8 cells were gated on living cells, whereas CD86+ (activated dendritic) cells were identified in the CD4-CD8- compartment. All data were acquired on a MACSQuant analyzer (Miltenyi Biotec, Bergisch Gladbach, Germany) and were analyzed with FlowJo Software v10.1 (Tree star, Ashland, OR, United States).

### Statistical Analysis

Medians, means, standard deviations (SD) and significance levels were determined using Mann–Whitney *U* test or one-way analysis of variance (ANOVA) with Tukey’s *post hoc* test for multiple comparisons (GraphPad Prism Software v6, La Jolla, CA, United States) as indicated. Two-sided probability (*p*) values ≤ 0.05 were considered significant. Experiments were reproduced twice and pooled data are shown (*n* = 8–15 per group).

## Results

### Kinetics of Intestinal Colonization Densities Following Recolonization of Secondary Abiotic Mice with *E. coli* or *L. johnsonii*

**FIGURE 1:**
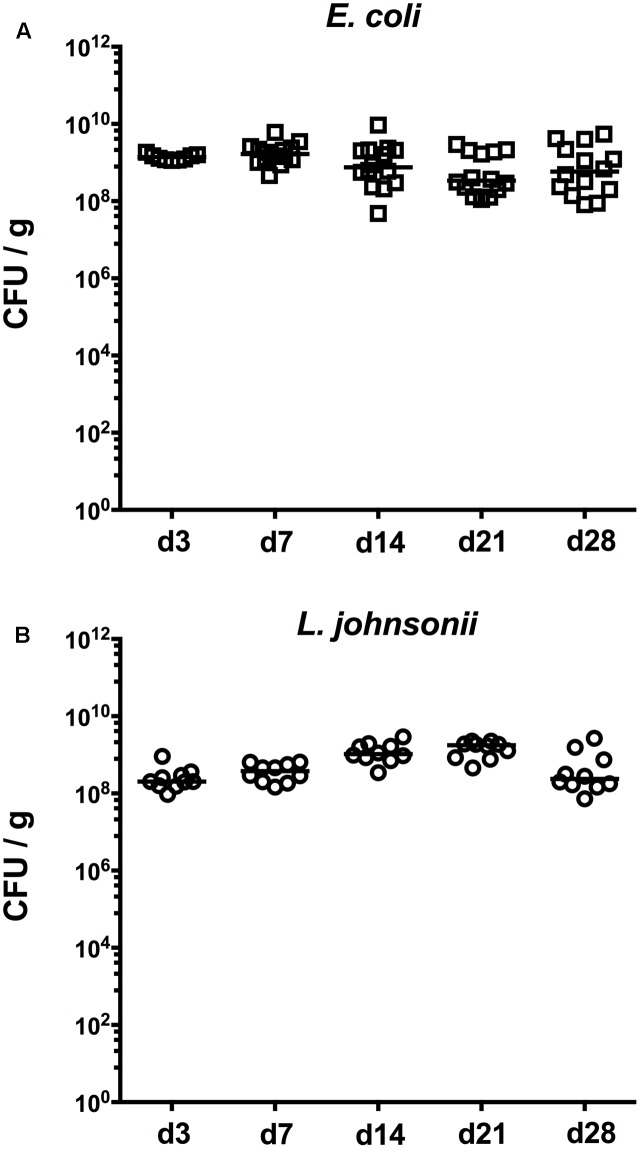
Kinetics of intestinal bacterial colonization densities following bacterial recolonization of secondary abiotic mice. Secondary abiotic mice were generated by broad-spectrum antibiotic treatment and perorally recolonized with **(A)** *E. coli* (open squares) or **(B)** *L. johnsonii* (open circles) on day (d) 0 as described in “Materials and Methods.” Bacterial colonization densities were assessed in fecal samples (colony forming units per gram, CFU/g) over time upon recolonization as indicated by culture. Medians (black bars) are indicated. Data were pooled from three independent experiments.

In the present study, we investigated the immunomodulatory properties of representative Gram-negative or Gram-positive intestinal bacterial species (namely, *E. coli* and *L. johnsonii*, respectively) in comparison to a complex gut microbiota in mice that had been treated with broad-spectrum antibiotic compounds. To address this, conventionally reared mice were subjected to a quintuple antibiotic cocktail rendering them secondary abiotic (Ekmekciu et al., 2017a,b), and after a sufficient wash-out period ABx mice were perorally recolonized with respective bacterial species or the complex intestinal microbiota by FMT. Importantly, the intestinal microbiota composition of ABx mice that had been subjected to FMT was comparable to naive conventionally colonized counterparts as confirmed previously (Ekmekciu et al., 2017a,b). As early as 3 days upon initial recolonization we assessed intestinal colonization efficiencies of respective species over time (**Figure 1**). Cultural analyses of fecal samples revealed that *E. coli* (**Figure 1A**) and *L. johnsonii* (**Figure 1B**) stably colonized the murine intestinal tract, both with high median loads of >10^8^ CFU per gram feces until necropsy at d28. Of note, none of the interventions carried out in this study (i.e., antibiotic treatment, recolonization with *E. coli*/*L. johnsonii*, FMT) led to any adverse clinical effects in mice such as wasting, diarrhea, occurrence of blood in feces or microscopic signs of intestinal inflammation including epithelial apoptosis (data not shown).

### T Cell Populations in Murine Intestinal and Systemic Compartments Following FMT or Recolonization of Secondary Abiotic Mice with *E. coli* or *L. johnsonii*

**FIGURE 2:**
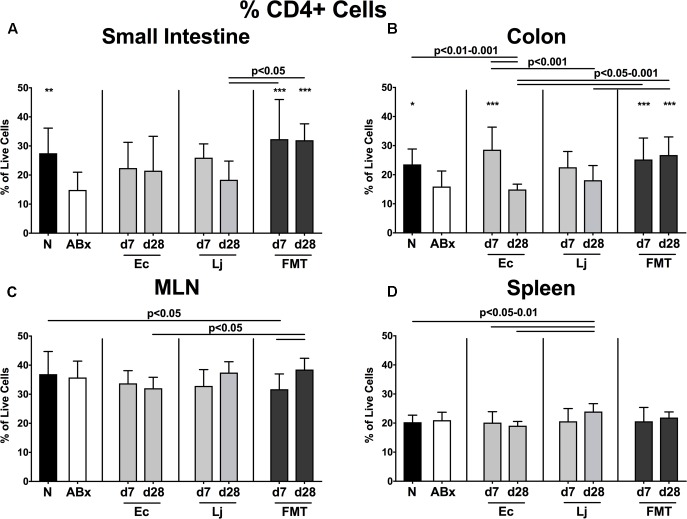
Percentages of CD4+ cells in intestinal and systemic compartments of secondary abiotic and recolonized mice. Secondary abiotic mice were generated by broad-spectrum antibiotic treatment and perorally recolonized by gavage. Subsequently, lymphocytes from small intestinal and colonic lamina propria, MLN and spleen were isolated, and analyzed by flow cytometry as described in “Materials and Methods.” The percentages of the CD4+ lymphocyte population within the **(A)** small intestine, **(B)** colon, **(C)** MLN, and **(D)** spleen of naive conventional mice (N), secondary abiotic mice (ABx) and mice re-associated with either *E. coli* (Ec), *L. johnsonii* (Lj) or complex intestinal microbiota by FMT on d7 and d28 post-recolonization are depicted. Columns represent means +SD. Significance levels (*p*-values) determined with one-way ANOVA test followed by Tukey post-correction test for multiple comparisons are indicated. Significant differences as compared to secondary abiotic mice are indicated by asterisks (^∗^*p* < 0.05; ^∗∗^*p* < 0.01; ^∗∗∗^*p* < 0.001). Data were pooled from three independent experiments.

**FIGURE 3:**
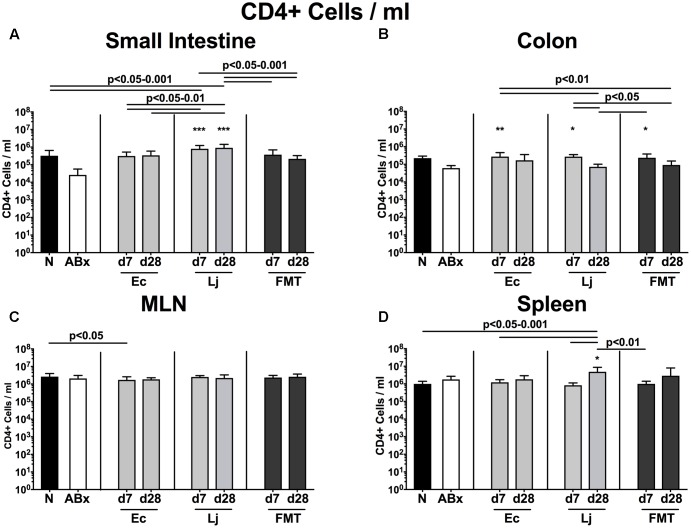
Absolute cell numbers of CD4+ cells in intestinal and systemic compartments of secondary abiotic and recolonized mice. Secondary abiotic mice were generated by broad-spectrum antibiotic treatment and perorally recolonized by gavage. Subsequently, lymphocytes from small intestinal and colonic lamina propria, MLN and spleen were isolated, and analyzed by flow cytometry as described in “Materials and Methods.” Concentrations of CD4+ lymphocytes in the **(A)** small intestine, **(B)** colon, **(C)** MLN, and **(D)** spleen of naive conventional mice (N), secondary abiotic mice (ABx) and mice re-associated with either *E. coli* (Ec), *L. johnsonii* (Lj) or complex intestinal microbiota by FMT on d7 and d28 post-recolonization are depicted. Columns represent means +SD. Significance levels (*p*-values) determined with one-way ANOVA test followed by Tukey post-correction test for multiple comparisons are indicated. Significant differences as compared to secondary abiotic mice are indicated by asterisks (^∗^*p* < 0.05; ^∗∗^*p* < 0.01; ^∗∗∗^*p* < 0.001). Data were pooled from three independent experiments.

To examine the impact of *E. coli* and *L. johnsonii* versus complex gut microbiota on distinct immune cell populations after antibiotic microbiota depletion, we isolated lymphocytes from small and large intestinal LP, MLN and spleen and analyzed defined immune cell populations by flow cytometry on d7 and d28 post recolonization. Firstly, we analyzed the CD4+ (**Figures 2**, **3**) and CD8+ lymphocytes (**Supplementary Figures S1**, **S2**) and assessed both the relative abundances (i.e., percentages) and the absolute cell numbers of respective immune cell populations. After the antibiotic treatment the abundance of CD4+ T helper lymphocytes declined in both the small and large intestines, but could be fully restored upon FMT (*p* < 0.05–0.001; **Figures 2A,B**). Mice harboring *E. coli* or *L. johnsonii* displayed slightly higher percentages of CD4+ cells in their small intestine without reaching significant levels (n.s. vs ABx; **Figure 2A**). In the colon, however, *E. coli* recolonization resulted in higher frequencies of CD4+ cells at d7, but decreased again until d28 (**Figure 2A**). Interestingly, in the MLN the percentage of the CD4+ lymphocytes was at its lowest at d7 post FMT (*p* < 0.05 vs. N; **Figure 2C**), but reached naive levels thereafter. In the spleens of *L. johnsonii* colonized mice we detected higher frequencies of CD4+ lymphocytes than in their with *E. coli* colonized counterparts at d28 (*p* < 0.05–0.01; **Figure 2D**). Furthermore, *L. johnsonii* recolonization led to the highest absolute CD4+ cell numbers in the murine small intestine (*p* < 0.05–0.001; **Figure 3A**). At d7 following either recolonization higher numbers of CD4+ lymphocytes could be observed in the colonic LP as compared to ABx mice (*p* < 0.05–0.01; **Figure 3B**), but declined until d28. Notably, at d28 after *L. johnsonii* colonization mice also displayed the highest numbers of CD4+ cells in the splenic (and hence systemic) compartment (*p* < 0.05–0.001; **Figure 3D**).

Interestingly, the microbiota depletion-induced decreases of the CD8+ cell abundances within the small and large intestinal LP were accompanied by increases of these cells in the spleen (*p* < 0.01–0.001 ABx vs. naive; **Supplementary Figures S1A,B,D**). Single strain mono-colonization could restore this cell population in the small intestine rather late, i.e., until d28 post recolonization with either *E. coli* or *L. johnsonii* (*p* < 0.05–0.001; **Supplementary Figure S1**). Remarkably, neither *E. coli* nor *L. johnsonii* colonization impacted the decline of colonic CD8+ cell numbers, which could only be restored upon FMT at d28 (**Supplementary Figure S1B**). At d7 post FMT, mice displayed the lowest frequencies of CD8+ cells in their MLN, which, however, increased until d28 (**Supplementary Figure S1C**). Moreover, at d7 following either recolonization, mice exerted lower percentages of splenic CD8+ cells than their ABx counterparts (*p* < 0.05–0.001; **Supplementary Figure S1D**). This effect, however, could be preserved over time in *E. coli* recolonized mice only. Remarkably, mice harboring single commensal species in their gut displayed higher CD8+ lymphocyte numbers in the small intestinal LP at d28 as compared to mice that had been subjected to FMT (*p* < 0.05–0.001; **Supplementary Figure S2A**). Until d7, however, only *L. johnsonii* but not *E. coli* could sufficiently restore the antibiotics-induced decreased colonic CD8+ lymphocytes (*p* < 0.01 vs. ABx; **Supplementary Figure S2B**). In line with the splenic CD4+ lymphocytes, the highest splenic CD8+ lymphocyte numbers could be detected in *L. johnsonii* colonized mice at d28 post recolonization (*p* < 0.001; **Supplementary Figure S2D**).

### Activated T Cells (Including Treg), Memory/Effector T Cells and Activated Dendritic Cells in Murine Intestinal and Systemic Compartments Following FMT or Recolonization of Secondary Abiotic Mice with *E. coli* or *L. johnsonii*

**FIGURE 4:**
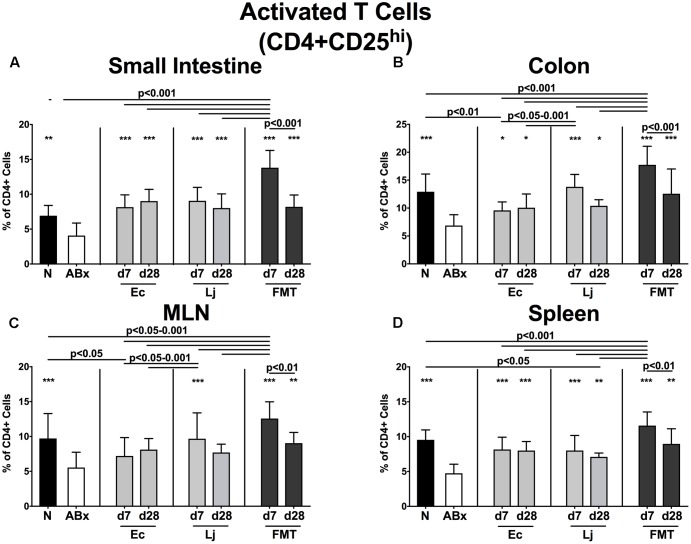
Activated T cells (including Treg) in intestinal and systemic compartments of secondary abiotic and recolonized mice. Secondary abiotic mice were generated by broad-spectrum antibiotic treatment and perorally recolonized by gavage. Subsequently, lymphocytes from small intestinal and colonic lamina propria, MLN and spleen were isolated, and analyzed by flow cytometry as described in “Materials and Methods.” The frequencies of activated T cells (including Treg, CD4+CD25+, gated on CD4+ cells) in the **(A)** small intestine, **(B)** colon, **(C)** MLN and **(D)** spleen of naive conventional mice (N), secondary abiotic mice (ABx) and mice re-associated with either *E. coli* (Ec), *L. johnsonii* (Lj) or complex intestinal microbiota by FMT on d7 and d28 post-recolonization are depicted. Columns represent means +SD. Significance levels (*p*-values) determined with one-way ANOVA test followed by Tukey post-correction test for multiple comparisons are indicated. Significant differences as compared to secondary abiotic mice are indicated by asterisks (^∗^*p* < 0.05; ^∗∗^*p* < 0.01; ^∗∗∗^*p* < 0.001). Data were pooled from three independent experiments.

**FIGURE 5:**
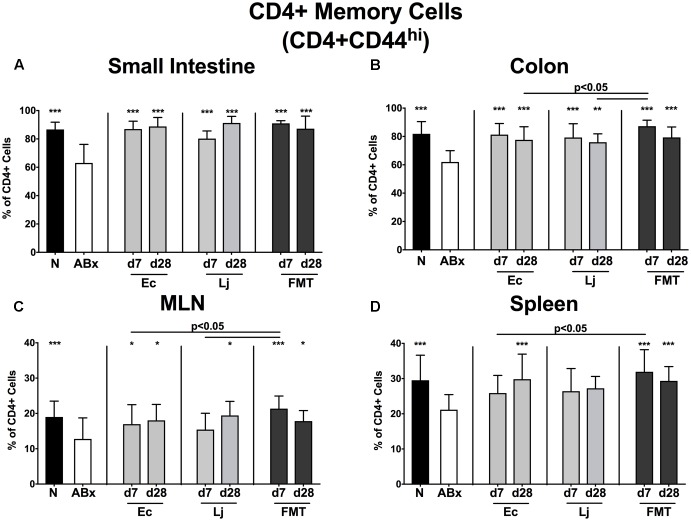
CD4+ memory/effector T cells in intestinal and systemic compartments of secondary abiotic and recolonized mice. Secondary abiotic mice were generated by broad-spectrum antibiotic treatment and perorally recolonized by gavage. Subsequently, lymphocytes from small intestinal and colonic lamina propria, MLN and spleen were isolated, and analyzed by flow cytometry as described in “Materials and Methods.” The proportions of CD4+ memory/effector cells (CD4+CD44^hi^, gated on CD4+ cells) in the **(A)** small intestine, **(B)** colon, **(C)** MLN and **(D)** spleen of naive conventional mice (N), secondary abiotic mice (ABx) and mice re-associated with either *E. coli* (Ec), *L. johnsonii* (Lj) or complex intestinal microbiota by FMT on d7 and d28 post-recolonization are depicted. Columns represent means +SD. Significance levels (*p*-values) determined with one-way ANOVA test followed by Tukey post-correction test for multiple comparisons are indicated. Significant differences as compared to secondary abiotic mice are indicated by asterisks (^∗^*p* < 0.05; ^∗∗^*p* < 0.01; ^∗∗∗^*p* < 0.001). Data were pooled from three independent experiments.

We further expanded our investigations by analyzing the activation status of defined immune cell populations. Therefore we stained for surface markers CD25 (**Figure 4**), CD44 (**Figure 5** and **Supplementary Figure S3**) and CD86 (**Supplementary Figure S4**) characteristic for activated T cells (including Treg), memory/effector T cells and activated DCs, respectively (Sprent and Surh, 2002; Wallet et al., 2005; Ostman et al., 2006). Broad-spectrum antibiotic treatment led to a significant reduction of the CD4+CD25+ abundances in all analyzed immunological compartments (**Figures 4A–D**). At d7 post FMT, mice displayed the highest percentages of CD4+CD25+ cells, which were even higher than in naive untreated SPF controls. However, both *E. coli* and *L. johnsonii* induced a significant increase of CD25 expression in lymphocytes derived from the small intestine, colon and spleen as early as d7 post recolonization (*p* < 0.05–0.001 vs. ABx; **Figures 4A,B,D**), whereas notably, *L. johnsonii* exerted a more pronounced effect than *E. coli* in the colon at d7 (*p* < 0.05–0.001 vs. Ec; **Figure 4B**). Moreover, only *L. johnsonii* was able to restore CD4+CD25+ cells in the MLN (*p* < 0.001 vs. ABx; **Figure 4C**).

Similarly, microbiota-depleted mice exhibited an overall reduction of the CD4+CD44+ memory/effector cell subset (*p* < 0.001 vs. N; **Figures 5A–D**). In the small and large intestine *E. coli*, *L. johnsonii* and FMT were equally capable of restoring this cell population as early as d7 post recolonization (*p* < 0.01–0.001 vs. ABx; **Figures 5A,B**). This also held true for the MLN, albeit in this compartment *L. johnsonii* could restore the abundances of CD4+CD44+ cells only rather late in the course, i.e., until d28 post recolonization. In contrast, *E. coli*, but not *L. johnsonii* colonization resulted in restored frequencies of splenic memory CD4+ cells at d28 post recolonization (*p* < 0.001 vs. ABx; **Figure 5D**).

Moreover, reduced abundances of CD8+CD44+ cells could be observed in the small intestine, colon, MLN and spleen of with antibiotics treated mice (*p* < 0.001 vs. N; **Supplementary Figures S3A–D**). Already at d7 following recolonization (regardless of the regimen) frequencies of CD8+ memory cells were elevated in the small intestine and spleen, whereas, in contrast to FMT, either single species colonization could normalize colonic CD8+ memory cells only until d28. In the MLN, *E. coli* was able to restore the CD8+ memory/effector cells more effectively than *L. johnsonii*, considering that abundances of CD8+CD44+ cells were comparable in *E. coli* mono-associated and naive mice (**Supplementary Figure S3C**).

The expression of the surface molecule CD86, a co-stimulatory protein marking activated DC, showed a strong intestinal microbiota dependence, as indicated by a strong reduction of CD86+ cells in mucosal and systemic compartments of secondary abiotic mice (*p* < 0.001 vs. N; **Supplementary Figures S4A–D**). Small intestinal CD86+ cells in mice harboring single bacterial species were more abundant than in ABx mice, however, lower than in their naive or with FMT treated counterparts (**Supplementary Figure S4A**). Moreover, only FMT, but neither *E. coli* nor *L. johnsonii*, could recover this antibiotics-induced reduction of CD86+ cell frequencies in the MLN until d28 (**Supplementary Figure S4C**). Splenic CD86+ cells displayed the highest abundance at d7 post FMT, but could also be restored to basal naive levels by either *E. coli* or *L. johnsonii* recolonization alone (*p* < 0.001 vs. ABx; **Supplementary Figure S4C**).

Taken together, our data indicate that depending on the respective immunological compartment and immune cell subset, single bacterial commensal species may be as effective as complex microbiota in reversing antibiotics induced collateral damages on intestinal mucosal and systemic immunity.

### Production of Pro- and Anti-inflammatory Cytokines by CD4+ Cells in Murine Intestinal and Systemic Compartments Following FMT or Recolonization of Secondary Abiotic Mice with *E. coli* or *L. johnsonii*

**FIGURE 6:**
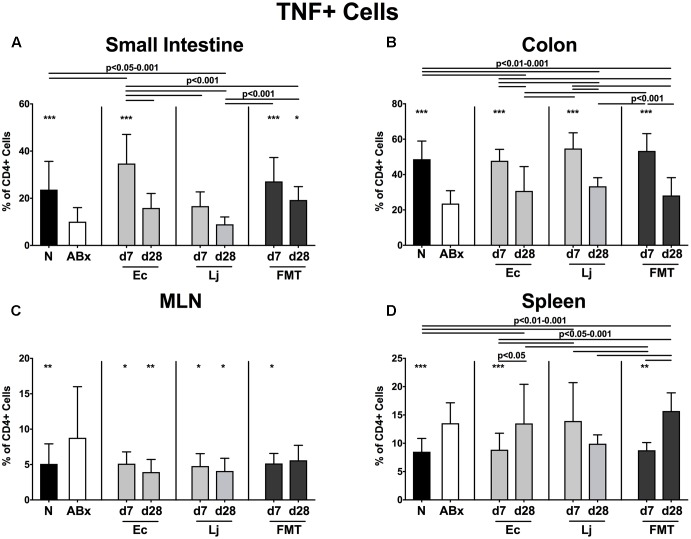
TNF producing CD4+ cells in intestinal and systemic compartments of secondary abiotic and recolonized mice. Secondary abiotic mice were generated by broad-spectrum antibiotic treatment and perorally recolonized by gavage. Subsequently, lymphocytes were isolated from small intestinal and colonic lamina propria, MLN, and spleen and stimulated with PMA/ionomycin in presence of brefeldin A and subsequently analyzed by flow cytometry. The percentages of IFN-γ producing CD4+ cells in the **(A)** small intestine, **(B)** colon, **(C)** MLN and **(D)** spleen of naive conventional mice (N), secondary abiotic mice (ABx) and mice re-associated with either *E. coli* (Ec), *L. johnsonii* (Lj) or complex intestinal microbiota by FMT on d7 and d28 post-recolonization are depicted. Columns represent means +SD. Significance levels (*p*-values) determined with one-way ANOVA test followed by Tukey post-correction test for multiple comparisons are indicated. Significant differences as compared to secondary abiotic mice are indicated by asterisks (^∗^*p* < 0.05; ^∗∗^*p* < 0.01; ^∗∗∗^*p* < 0.001). Data were pooled from three independent experiments.

**FIGURE 7:**
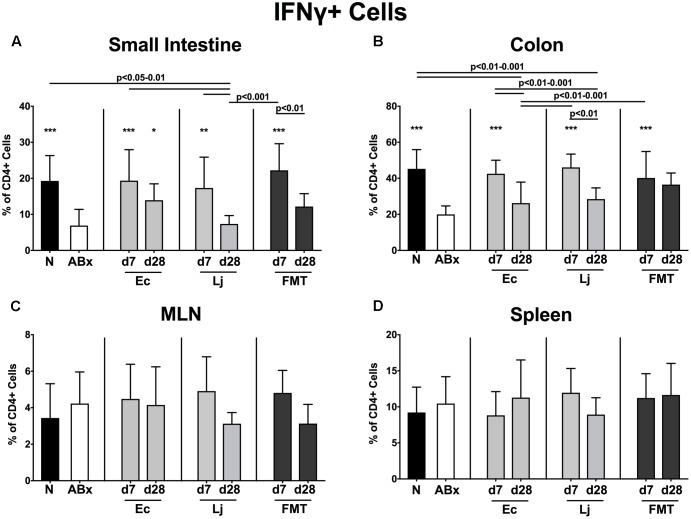
IFN-γ producing CD4+ cells in intestinal and systemic compartments of secondary abiotic and recolonized mice. Secondary abiotic mice were generated by broad-spectrum antibiotic treatment and perorally recolonized by gavage. Subsequently, lymphocytes were isolated from small intestinal and colonic lamina propria, MLN, and spleen and stimulated with PMA/ionomycin in presence of brefeldin A and subsequently analyzed by flow cytometry. The percentages of TNF producing CD4+ cells in the **(A)** small intestine, **(B)** colon, **(C)** MLN and **(D)** spleen of naive conventional mice (N), secondary abiotic mice (ABx) and mice re-associated with either *E. coli* (Ec), *L. johnsonii* (Lj) or complex intestinal microbiota by FMT on d7 and d28 post-recolonization are depicted. Columns represent means +SD. Significance levels (*p*-values) determined with one-way ANOVA test followed by Tukey post-correction test for multiple comparisons are indicated. Significant differences as compared to secondary abiotic mice are indicated by asterisks (^∗^*p* < 0.05; ^∗∗^*p* < 0.01; ^∗∗∗^*p* < 0.001). Data were pooled from three independent experiments.

**FIGURE 8:**
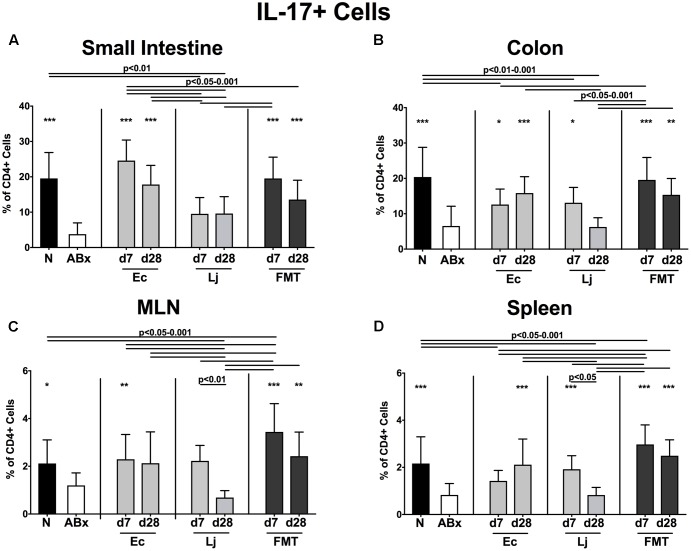
IL-17 producing CD4+ cells in intestinal and systemic compartments of secondary abiotic and recolonized mice. Secondary abiotic mice were generated by broad-spectrum antibiotic treatment and perorally recolonized by gavage. Subsequently, lymphocytes were isolated from small intestinal and colonic lamina propria, MLN, and spleen and stimulated with PMA/ionomycin in presence of brefeldin A and subsequently analyzed by flow cytometry. The percentages of IL-17 producing CD4+ cells in the **(A)** small intestine, **(B)** colon, **(C)** MLN and **(D)** spleen of naive conventional mice (N), secondary abiotic mice (ABx) and mice re-associated with either *E. coli* (Ec), *L. johnsonii* (Lj) or complex intestinal microbiota by FMT on d7 and d28 post-recolonization are depicted. Columns represent means +SD. Significance levels (*p*-values) determined with one-way ANOVA test followed by Tukey post-correction test for multiple comparisons are indicated. Significant differences as compared to secondary abiotic mice are indicated by asterisks (^∗^*p* < 0.05; ^∗∗^*p* < 0.01; ^∗∗∗^*p* < 0.001). Data were pooled from three independent experiments.

**FIGURE 9:**
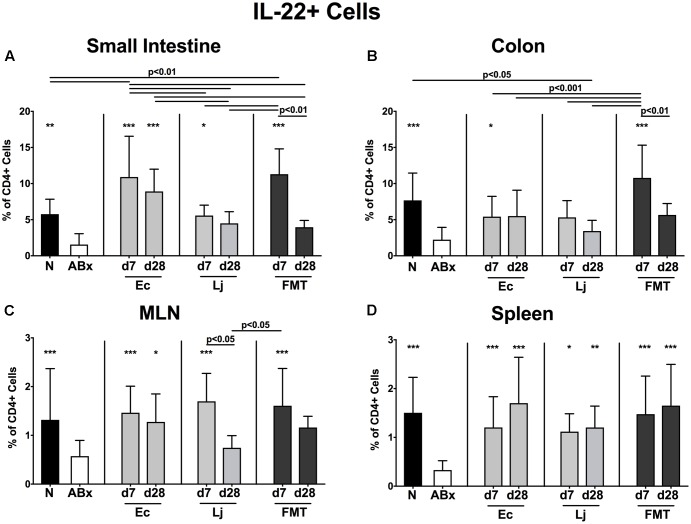
IL-22 producing CD4+ cells in intestinal and systemic compartments of secondary abiotic and recolonized mice. Secondary abiotic mice were generated by broad-spectrum antibiotic treatment and perorally recolonized by gavage. Subsequently, lymphocytes were isolated from small intestinal and colonic lamina propria, MLN, and spleen and stimulated with PMA/ionomycin in presence of brefeldin A and subsequently analyzed by flow cytometry. The percentages of IL-22 producing CD4+ cells in the **(A)** small intestine, **(B)** colon, **(C)** MLN and **(D)** spleen of naive conventional mice (N), secondary abiotic mice (ABx) and mice re-associated with either *E. coli* (Ec), *L. johnsonii* (Lj) or complex intestinal microbiota by FMT on d7 and d28 post-recolonization are depicted. Columns represent means +SD. Significance levels (*p*-values) determined with one-way ANOVA test followed by Tukey post-correction test for multiple comparisons are indicated. Significant differences as compared to secondary abiotic mice are indicated by asterisks (^∗^*p* < 0.05; ^∗∗^*p* < 0.01; ^∗∗∗^*p* < 0.001). Data were pooled from three independent experiments.

**FIGURE 10:**
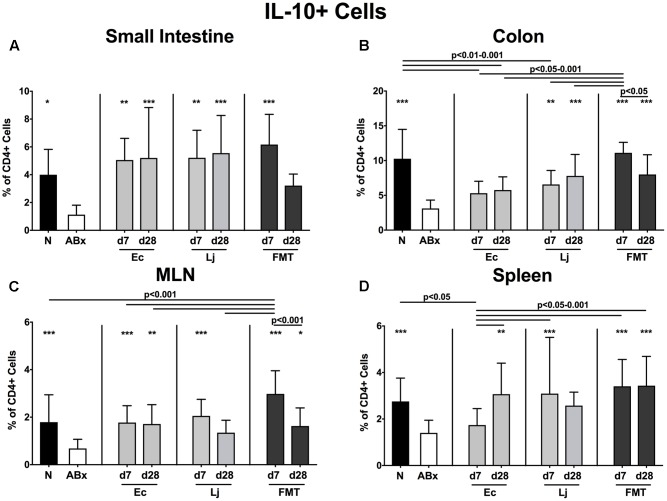
IL-10 producing CD4+ cells in intestinal and systemic compartments of secondary abiotic and recolonized mice. Secondary abiotic mice were generated by broad-spectrum antibiotic treatment and perorally recolonized by gavage. Subsequently, lymphocytes were isolated from small intestinal and colonic lamina propria, MLN, and spleen and stimulated with PMA/ionomycin in presence of brefeldin A and subsequently analyzed by flow cytometry. The percentages of IL-10 producing CD4+ cells in the **(A)** small intestine, **(B)** colon, **(C)** MLN and **(D)** spleen of naive conventional mice (N), secondary abiotic mice (ABx) and mice re-associated with either *E. coli* (Ec), *L. johnsonii* (Lj) or complex intestinal microbiota by FMT on d7 and d28 post-recolonization are depicted. Columns represent means +SD. Significance levels (*p*-values) determined with one-way ANOVA test followed by Tukey post-correction test for multiple comparisons are indicated. Significant differences as compared to secondary abiotic mice are indicated by asterisks (^∗^*p* < 0.05; ^∗∗^*p* < 0.01; ^∗∗∗^*p* < 0.001). Data were pooled from three independent experiments.

We further addressed the impact of *E. coli* and *L. johnsonii* recolonization on the cytokine production pattern of CD4+ lymphocytes in intestinal mucosal, peripheral and systemic immunological sites and therefore determined the frequencies of TNF (**Figure 6**), IFN-γ (**Figure 7**), IL-17 (**Figure 8**), IL-22 (**Figure 9**), and IL-10 (**Figure 10**) producing CD4+ lymphocytes in the small and large intestines, MLN and spleens of recolonized mice.

The antibiotics-induced decreases of TNF producing CD4+ cells in the small and large intestines were accompanied by increased abundances in the MLN and spleen (*p* < 0.01–0.001 ABx vs. N; **Figures 6A–D**). Small intestinal TNF production was at it highest at d7 following *E. coli* recolonization, but further declined thereafter, while it remained unaffected by *L. johnsonii* during the entire observation period. In addition, either recolonization regimen resulted in higher abundances of TNF producing CD4+ cells in the colonic LP at d7, whereas none of them could sustain this effect until d28. Of note, microbial reassociation of ABx mice dampened TNF production in the MLN (*p* < 0.05–0.01 vs. ABx; **Figure 6C**), except for FMT at d28. *E. coli* and complex microbiota could additionally reduce the TNF production in the spleen at d7 (*p* < 0.01–0.001 vs. ABx, n.s. vs. N; **Figure 6D**).

Furthermore, a significant reduction of IFN-γ producing CD4+ cells could be detected in the small and large intestines of secondary abiotic mice (*p* < 0.001 vs. N; **Figures 7A,B**). *E. coli* at d7 and d28, as well as *L. johnsonii* and FMT at d7 could completely restore the IFN-γ production in the small intestine. Colonic IFN-γ expressing CD4+ cells also increased upon either recolonization regimen at d7 (*p* < 0.001; **Figure 7B**). In contrast, IFN-γ production remained largely unaffected by antibiotic treatment and subsequent bacterial reassociation in the MLN and spleen of mice (**Figures 7C,D**).

Furthermore, IL-17 expressing CD4+ cells were strongly diminished in all analyzed immunological compartments upon microbial depletion, but could be fully restored by FMT (*p* < 0.05–0.001; **Figures 8A–D**). Mono-association with *E. coli* resulted in elevated IL-17 production in the small intestine and colon at both d7 and d28, as well as in the MLN at d7 and spleen at d28 (**Figures 8A–D**). Mice harboring *L. johnsonii* alone displayed higher abundances of CD4+IL17+ cells than ABx mice in the colon and spleen at d7 post recolonization. In addition, decreased percentages of IL-22 producing CD4+ cells following broad-spectrum antibiotic treatment were detected in either compartment (*p* < 0.01–0.001; **Figures 9A–D**). Small intestinal IL-22 expression was most distinctly induced by *E. coli* as early as d7 and by FMT at d7 only (**Figure 9A**). Mono-associated mice further displayed slightly higher abundances of colonic IL-22 producing CD4+ cells than their ABx counterparts, which were, however, only significantly higher in the case of *E. coli* at d7 (*p* < 0.05 vs. ABx; **Figure 9B**). Either recolonization regimen could restore the CD4+IL22+ immune cell subset in the MLN at d7, but only *E. coli* was able to sustain this effect until d28. Moreover, recolonized mice showed similar frequencies of IL-22 producing CD4+ lymphocytes in the systemic compartment as their naive, untreated counterparts, regardless of the intervention or time point (*p* < 0.05–0.001; **Figures 9C,D**).

Overall, the IL-10 expressing CD4+ lymphocytes were down-regulated in the absence of the intestinal microbiota (*p* < 0.05–0.001; **Figures 10A–D**). Both *E. coli* and *L. johnsonii* mono-colonization, however, could sufficiently restore the CD4+IL10+ cells in the small intestines, whereas only *L. johnsonii* could achieve this effect in the colon (**Figures 10A,B**). Increased abundances of IL-10 producing CD4+ cells were also detected upon *E. coli* recolonization at d7 in the MLN and d28 in both MLN and spleen. The IL-10 production in the MLN and spleen could also be restored at d7 following *L. johnsonii* recolonization, but this effect could not be sustained until d28 post recolonization.

## Discussion

In the present study, we aimed at comparing the short- and long-term effects of a single representative Gram-negative and Gram-positive intestinal commensal species, namely of *E. coli* and *L. johnsonii*, respectively, versus the complex gut microbiota in shaping host immunity following antibiotics-induced microbiota depletion. These species were chosen for the following reasons. Firstly, both commensals are common inhabitants of the intestinal ecosystem, and although lactobacilli are quantitatively more abundant than *E. coli* (Castillo et al., 2006), the latter represents the predominant facultative anaerobe Gram-negative strain within the mammalian gastrointestinal tract (Finegold et al., 1983; Tenaillon et al., 2010). Furthermore, in our previous works we could confirm differential immunomodulatory properties of either strain under defined immunopathological conditions. For instance, *E. coli*, but not *L. johnsonii*, aggravated Th1-driven pro-inflammatory immune responses in murine ileitis (Heimesaat et al., 2006, 2007). Furthermore, *L. johnsonii* was able to attenuate intestinal and systemic pro-inflammatory and to augment anti-inflammatory immune responses upon *C. jejuni* infection of secondary abiotic mice (Bereswill et al., 2017), and has further been shown to be effective against enteric including infectious morbidities (Lievin-Le Moal and Servin, 2014), hence resulting in its commercial probiotic application (e.g., Nestlé LC1).

Here, we unraveled immunomodulatory properties of respective commensals at two different time points following recolonization of antibiotics-treated mice, but in the absence of immunopathological conditions. Performing kinetic analysis in this context is important, given that intestinal responses are highly dynamic over time after the transfer of microbiota into germ-free animals (El Aidy et al., 2012; Tomas et al., 2015).

Cultural analysis of fecal samples revealed that either commensal strain was able to stably colonize the murine intestinal tract at high intestinal loads throughout the observation period. Our previous work revealed that upon cessation of antibiotic therapy neither regrowth of intestinal bacterial commensals nor changes in the immune cell populations could be observed, further supporting that the observed immune responses of the mucosal and systemic sites can be exclusively attributed to defined bacterial commensal recolonization (Ekmekciu et al., 2017a,b).

Overall, single-strain recolonization appeared to be, to some extent, less effective in restoring immune cell populations after broad-spectrum antibiotic treatment than the complex microbiota upon FMT, given that mono-colonized mice displayed a lack of full recovery of affected cell populations. Following mono-colonization of mice both common and strict strain-specific immune responses could be observed over time. For instance, *E. coli* and *L. johnsonii* had a minor impact on the relative abundances of intestinal CD4+ and CD8+ cells when compared to antibiotics-treated mice, while FMT resulted in cell percentages comparable to naive conventionally colonized counterparts. Furthermore, *E. coli*, but not *L. johnsonii* recolonization could increase colonic CD4+ abundances at d7, but not later on. Whereas small intestinal CD8+ cells could be reestablished only rather late upon mono-colonization, colonic CD8+ cells were virtually unaffected. In the systemic compartment, however, the observed increases in percentages of splenic CD8+ cells in microbiota-depleted mice could be reversed by both *E. coli* and *L. johnsonii* until d7, and remained lower in *E. coli* recolonized mice later on. These results underline the importance of examining immunological sequelae of gut microbiota-interventive strategies, not only at mucosal surfaces, but also on the systemic level of the immune system, given that the presence/absence and distinct composition of the gut microbiota might also affect splenic lymphocytes. This rationale is supported by the former findings that the *Bacteroides fragilis* driven differentiation of Treg was not limited to the LP, but could also be detected in the circulation (Round et al., 2011).

Interestingly, recolonization with *L. johnsonii* resulted in highest CD4+ lymphocyte numbers in the small intestinal LP, supporting former evidence from a clinical investigation, where the application of another *Lactobacillus* strain, namely *L. reuteri*, led to a significant increase in ileal CD4+ cells (Valeur et al., 2004). In our study, either commensal strain was able to promote CD25 expression on CD4+ lymphocytes in the small and large intestines as well as in the spleen, but only *L. johnsonii* was able to achieve the same effect in MLN. Of note, at d7 following recolonization, *L. johnsonii* was a stronger inducer of the colonic activated T cells (including Treg) than *E. coli*. The dependence of Treg on intestinal bacterial antigens has been demonstrated before. Atarashi et al. (2011) showed that treatment of mice with vancomycin, an antibiotic compound directed mainly against Gram-positive bacteria, resulted in reduced numbers of colonic Treg (Zarkan et al., 2016). A study conducted with germ-free rats revealed that colonic CD25+ cells were higher in animals co-colonized with *E. coli* and *L. plantarum* 299v, than in those harboring *E. coli* alone (Herías et al., 1999). This indicates that increased microbiota diversity and synergistic effects between species may elicit a stronger immunomodulatory effect on activated T cells and the Treg population, which is supported by our findings that FMT induced the most prominent expression of CD25 in all analyzed immunological compartments.

Unlike FMT, single-strain recolonization resulted in an only partial restoration of the intestinal activated DC (CD86+) cells, which remained, however, lower than in untreated SPF mice. Former evidence suggests, that the activation, maturation and cytokine production of DC upon bacterial stimulation is highly dose dependent (Evrard et al., 2011). Our data indicate that the activation of DC may furthermore be dependent on the diversity of presented microbial antigens.

*In vitro* studies applying PBMC revealed that Gram-positive and Gram-negative bacteria differentially induced cytokine expression patterns (Hessle et al., 2000, 2005; Skovbjerg et al., 2010). Therefore we analyzed the production of pro- and anti-inflammatory cytokines, including TNF, IFN-γ, IL-17, IL-22, and IL-10 in mice following recolonization with either *E. coli* or *L. johnsonii*. Intestinal *E. coli* colonization had a more prominent effect on TNF production by lymphocytes than *L. johnsonii*. At d7 following *E. coli* application, TNF levels measured in intestinal, peripheral and systemic immune compartments were comparable to those in naive mice. However, this effect could be preserved until d28 in MLN only. While excessive TNF production has conclusively been linked to chronic inflammation in numerous organs including the gastrointestinal tract (Kollias et al., 1999; Popa et al., 2007), it is important to recognize the protective roles of TNF against epithelial injury and disease susceptibility. In a murine ileitis model increased TNF levels resulted in improved epithelial barrier functions and prevention from disease onset (Pagnini et al., 2009). Moreover, TNF deficient mice were more prone to acute colitis, thus leading to the conclusion that TNF might have protective functions in normal gut homeostasis and intestinal epithelial integrity (Naito et al., 2003). Rakoff-Nahoum et al. (2004) suggested that the production of intestinal epithelial tissue-protective factors, including TNF, is regulated via TLR-mediated recognition of commensal bacteria, while we here provide further evidence that the adaptive immune system may also contribute to this effect.

Furthermore, both *E. coli* and *L. johnsonii* were able to promote the small intestinal and colonic production of IFN-γ by CD4+ cells as early as d7 post recolonization, whereas this cytokine remained highly expressed until d28 only in the small intestines of *E. coli* recolonized mice. The abundance of IFN-γ expressing CD4+ lymphocytes in the MLN and spleen remained unaffected by any of the interventions. These data seem to contradict *in vitro* findings, which suggest that Gram-positive bacteria tend to be stronger inducers of IFN-γ and TNF than Gram-negative species (Hessle et al., 2000, 2005; Skovbjerg et al., 2010). Apart from the general difficulty of directly applying *in vitro* results to vertebrates exhibiting a complex intestinal microbiota and distinct intra-luminal milieu interacting with the fine-tuned immune system in health and disease, it is also highly likely that human PBMC and murine CD4+ cells differently respond to bacterial stimuli, given former evidence regarding the differences in cytokine patterns between human monocytes and human DC upon bacterial stimulation (Karlsson et al., 2004).

Moreover, *E. coli* recolonization seemed to favor a Th17 immune cell differentiation, particularly in the small intestine, given that the production of the two key cytokines of this cell population, i.e., IL-17 and IL-22, was significantly higher in CD4+ cells isolated from with *E. coli* recolonized mice as compared to mice harboring *L. johnsonii* only. Th17 polarization is determined by the co-stimulation and cytokine provision from the DC (Perona-Wright et al., 2009), whereby IL-23 plays a particularly important role in the sustenance of Th17 cell responses *in vivo* (Mangan et al., 2006; McGeachy et al., 2009). It is tempting to speculate that commensal *E. coli* more strongly induce IL-23 production of DC, thus leading to a Th17 cell differentiation. Interestingly, a study conducted with human PBMC revealed that myeloid DC do indeed produce higher levels of IL-23 when stimulated with *E. coli* (Manuzak et al., 2012). Furthermore, *E. coli* heat-labile enterotoxin (synergizing with LPS) has been shown to induce IL-1β and IL-23 secretion by DC, which in turn promoted IL-17 and IFN-γ production by CD4+ T cells (Brereton et al., 2011).

*Lactobacillus johnsonii* recolonized mice, however, displayed higher abundances of CD4+IL-17+ cells in the colon and spleen and of CD4+IL22+ cells in the small intestinal LP and MLN as compared to their secondary abiotic counterparts at d7 post recolonization. Nonetheless, all the aforementioned immune cell populations declined back to the levels observed in secondary abiotic mice until 4 weeks after *L. johnsonii* recolonization. It has been known for a while now, that specific bacterial species such as SFB are required for the differentiation of small intestinal Th17 cells in germ-free mice (Ivanov et al., 2008; Gaboriau-Routhiau et al., 2009). A more recent investigation revealed that extracellular pathogens, such as *Citrobacter rodentium* and *E. coli* O157:H7 can also induce Th17 differentiation following adhesion to intestinal epithelial cells (Atarashi et al., 2015). Importantly, a common pilus adherence factor, which mediates adhesion to epithelial cells, has been reported for both pathogenic and commensal *E. coli* species (Rendón et al., 2007), indicating that the observed effects on the Th17 cells may be mediated through adherence of this commensal *E. coli*.

Furthermore, both *E. coli* and *L. johnsonii* could restore the antibiotics-induced reduction of IL-10 producing CD4+ cells in the small intestines, but only the latter could achieve the same effect in the colon, indicating commensal species-specific anti-inflammatory properties. Our results are well in line with a comprehensive analysis of numerous *Lactobacillus* species suggesting that species derived from healthy mice can be largely classified as potentially anti-inflammatory (Gorska et al., 2016). Notably, these data support the already proposed concept and evidence, that probiotic bacteria including *L. johnsonii* exert health-promoting effects through induction of regulatory/anti-inflammatory host immune responses (Di Giacinto et al., 2005; Kwon et al., 2010). However, it is highly likely that *L. johnsonii* and other probiotic species exert additional beneficial functions through IL-10 independent mechanisms, given their capability to attenuate the severity of colonic inflammation in IL-10 deficient mice.

The reintroduction of complex microbiota into the host via FMT is a well-known therapy dating back to the Chinese Dong-jin dynasty in the fourth century (Zhang et al., 2012) and has undergone a renaissance recently as a therapeutic option for the treatment of recurrent and refractory *Clostridium difficile* toxin induced acute necrotizing pseudo-membranous enterocolitis (van Nood et al., 2009; Rohlke et al., 2010; Brandt et al., 2012; Fischer et al., 2016; Scaldaferri et al., 2016).

In summary, the restoration of immune cell populations following broad-spectrum antibiotic treatment by commensal bacterial mono-colonization appears to be less effective than by complex intestinal microbiota association upon FMT, but depends both on the respective immune cell subtypes and analyzed compartments and does hence not follow a clear cut pattern. Nevertheless, mono-colonization of secondary abiotic mice with defined Gram-negative or Gram-positive species has a deep impact on shaping the host immune system and drives both pro- as well as anti-inflammatory immune responses on the mucosal, peripheral and systemic level in parallel. However, *E. coli* appears to favor pro-inflammatory immune responses, while *L. johnsonii* has a stronger impact on anti-inflammatory immune cell subsets.

## Conclusion

Future studies should further unravel the fine-tuned interplay between the well-orchestrated concert within the complex intestinal microbiota intestinal on one side and the different members of the immune system on the other in health and disease subsequently providing potential novel treatment options for immunopathological intestinal and extra-intestinal morbidities.

## Author Contributions

IE: Performed experiments, analyzed data, and wrote paper. EvK: Performed experiments. CN: Suggested critical parameters in design of experiments, supplied antibodies. PB: Suggested critical parameters in design of experiments, supplied antibodies. AS: Provided advice in design and performance of experiments. SB: Provided advice in design and performance of experiments, co-edited paper. MH: Designed and performed experiments, analyzed data, and co-wrote paper.

## Conflict of Interest Statement

The authors declare that the research was conducted in the absence of any commercial or financial relationships that could be construed as a potential conflict of interest.

## Supplementary Material

**FIGURE S1** | Percentages of CD8+ cells in intestinal and systemic compartments of secondary abiotic and recolonized mice. Secondary abiotic mice were generated by broad-spectrum antibiotic treatment and perorally recolonized by gavage. Subsequently, lymphocytes from small intestinal and colonic lamina propria, MLN and spleen were isolated, and analyzed by flow cytometry as described in “Materials and Methods.” The percentages of the CD8+ lymphocyte population within the **(A)** small intestine, **(B)** colon, **(C)** MLN and **(D)** spleen of naive conventional mice (N), secondary abiotic mice (ABx) and mice re-associated with either *E. coli* (Ec), *L. johnsonii* (Lj) or complex intestinal microbiota by FMT on d7 and d28 post-recolonization are depicted. Columns represent means +SD. Significance levels (*p*-values) determined with one-way ANOVA test followed by Tukey post-correction test for multiple comparisons are indicated. Significant differences as compared to secondary abiotic mice are indicated by asterisks (^∗^*p* < 0.05; ^∗∗^*p* < 0.01; ^∗∗∗^*p* < 0.001). Data were pooled from three independent experiments.

**FIGURE S2** | Absolute cell numbers of CD8+ cells in intestinal and systemic compartments of secondary abiotic and recolonized mice. Secondary abiotic mice were generated by broad-spectrum antibiotic treatment and perorally recolonized by gavage. Subsequently, lymphocytes from small intestinal and colonic lamina propria, MLN and spleen were isolated, and analyzed by flow cytometry as described in “Materials and Methods.” The concentrations of CD8+ lymphocytes in the **(A)** small intestine, **(B)** colon, **(C)** MLN and **(D)** spleen of naive conventional mice (N), secondary abiotic mice (ABx) and mice re-associated with either *E. coli* (Ec), *L. johnsonii* (Lj) or complex intestinal microbiota by FMT on d7 and d28 post-recolonization are depicted. Columns represent means +SD. Significance levels (*p*-values) determined with one-way ANOVA test followed by Tukey post-correction test for multiple comparisons are indicated. Significant differences as compared to secondary abiotic mice are indicated by asterisks (^∗^*p* < 0.05; ^∗∗^*p* < 0.01; ^∗∗∗^*p* < 0.001). Data were pooled from three independent experiments.

**FIGURE S3** | CD8+ memory/effector T cells in intestinal and systemic compartments of secondary abiotic and recolonized mice. Secondary abiotic mice were generated by broad-spectrum antibiotic treatment and perorally recolonized by gavage. Subsequently, lymphocytes from small intestinal and colonic lamina propria, MLN and spleen were isolated, and analyzed by flow cytometry as described in “Materials and Methods.” The proportions of CD8+ memory/effector cells (CD8+CD44^hi^, gated on CD8+ cells) in the **(A)** small intestine, **(B)** colon, **(C)** MLN and **(D)** spleen of naive conventional mice (N), secondary abiotic mice (ABx) and mice re-associated with either *E. coli* (Ec), *L. johnsonii* (Lj) or complex intestinal microbiota by FMT on d7 and d28 post-recolonization are depicted. Columns represent means +SD. Significance levels (*p*-values) determined with one-way ANOVA test followed by Tukey post-correction test for multiple comparisons are indicated. Significant differences as compared to secondary abiotic mice are indicated by asterisks (^∗^*p* < 0.05; ^∗∗^*p* < 0.01; ^∗∗∗^*p* < 0.001). Data were pooled from three independent experiments.

**FIGURE S4** | Activated DCs in intestinal and systemic compartments of secondary abiotic and recolonized mice. Secondary abiotic mice were generated by broad-spectrum antibiotic treatment and perorally recolonized by gavage. Subsequently, lymphocytes from small intestinal and colonic lamina propria, MLN and spleen were isolated, and analyzed by flow cytometry as described in “Materials and Methods.” The frequencies of activated DCs (CD86+, gated on CD4-CD8- live cells) in the **(A)** small intestine, **(B)** colon, **(C)** MLN and **(D)** spleen of naive conventional mice (N), secondary abiotic mice (ABx) and mice re-associated with either *E. coli* (Ec), *L. johnsonii* (Lj) or complex intestinal microbiota by FMT on d7 and d28 post-recolonization are depicted. Columns represent means +SD. Significance levels (*p*-values) determined with one-way ANOVA test followed by Tukey post-correction test for multiple comparisons are indicated. Significant differences as compared to secondary abiotic mice are indicated by asterisks (^∗^*p* < 0.05; ^∗∗^*p* < 0.01; ^∗∗∗^*p* < 0.001). Data were pooled from three independent experiments.

## Abbreviations

ABx: secondary abiotic
BSA: bovine serum albumin
CFU: colony forming units
DC: dendritic cells
FMT: fecal microbiota transplantation
IFN-γ: interferon-γ
IL: interleukin
LPL: lamina propria lymphocytes
LPS: lipopolysaccharide
MLN: mesenteric lymph nodes
PBS: phosphate buffered saline
PBMC: peripheral blood mononuclear cells
PP: Peyer’s patches
PSA: polysaccharide A
SFB: segmented filamentous bacteria
SPF: special pathogen free
Th: T helper cell
TLR: toll-like receptor
TNF: tumor necrosis factor
Treg: regulatory T cells
