# Supplementary figures and images for: Fecal Microbiota Transplantation, Commensal Escherichia coli and Lactobacillus johnsonii Strains Differentially Restore Intestinal and Systemic Adaptive Immune Cell Populations Following Broad-spectrum Antibiotic Treatment
Source: Front Microbiol. 2017 Dec 11;8:2430. doi: 10.3389/fmicb.2017.02430 (PMC5732213; doi:10.3389/fmicb.2017.02430)

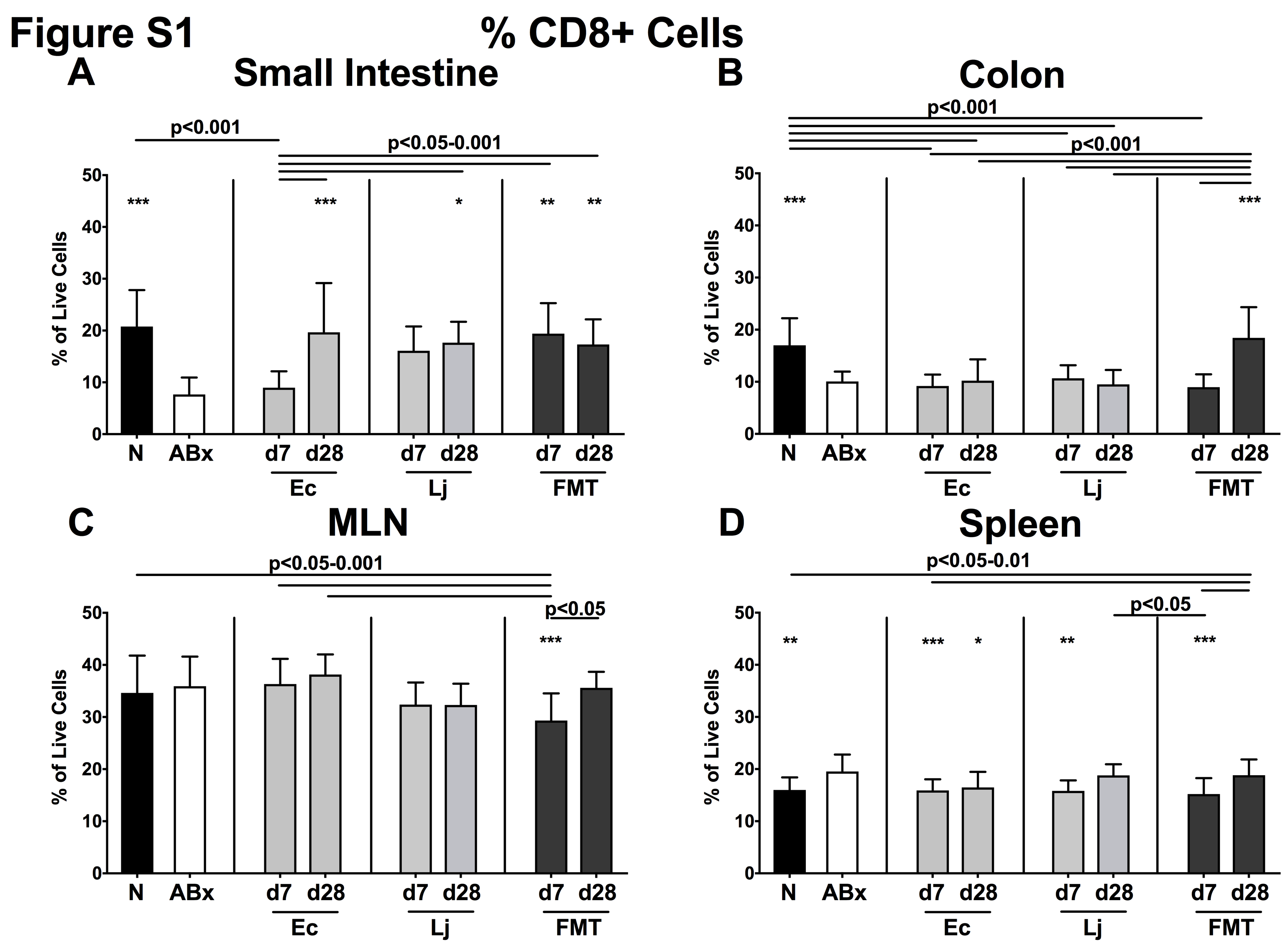

Supplement: Supplementary file 1 [file Image_1.TIFF]

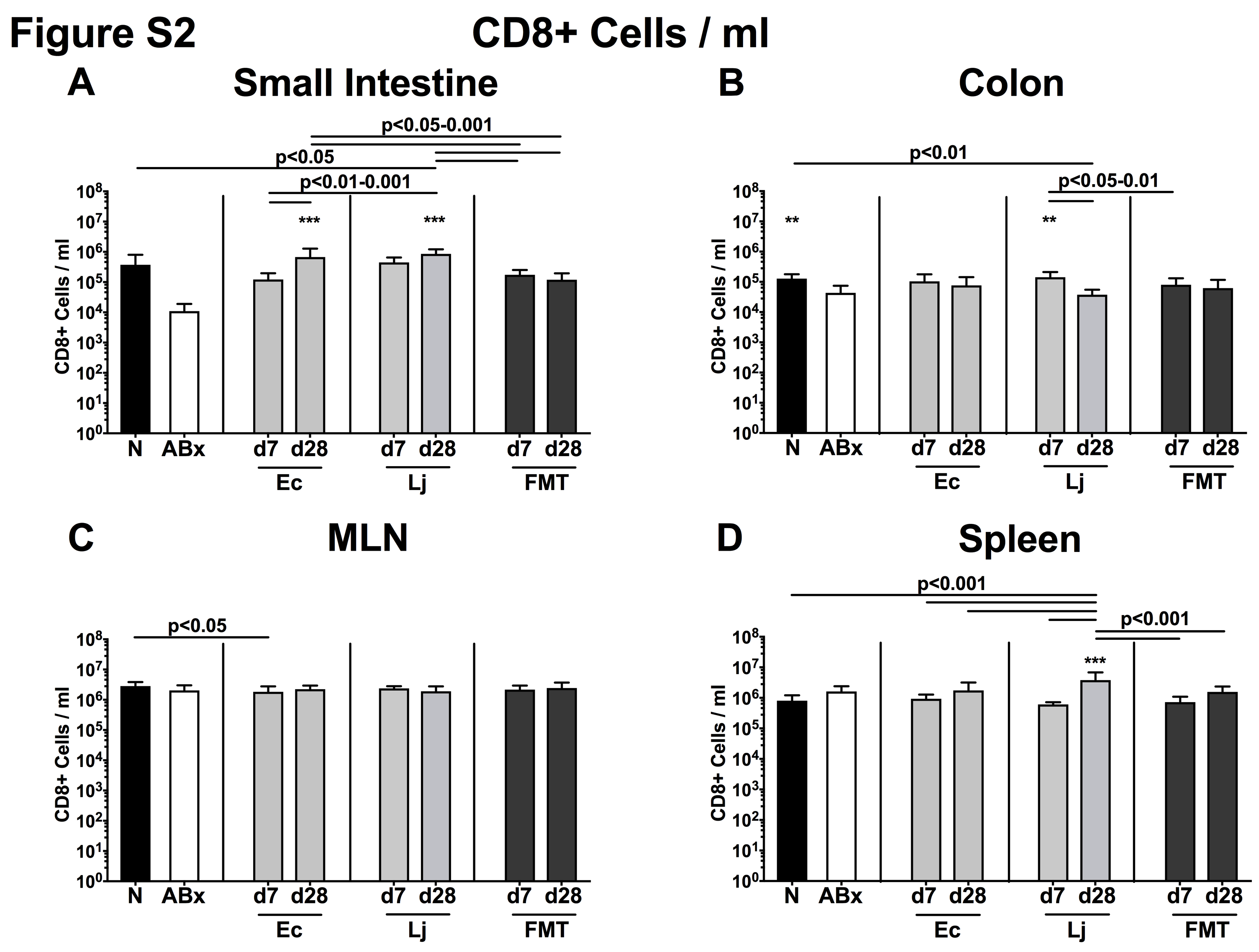

Supplement: Supplementary file 2 [file Image_2.TIFF]

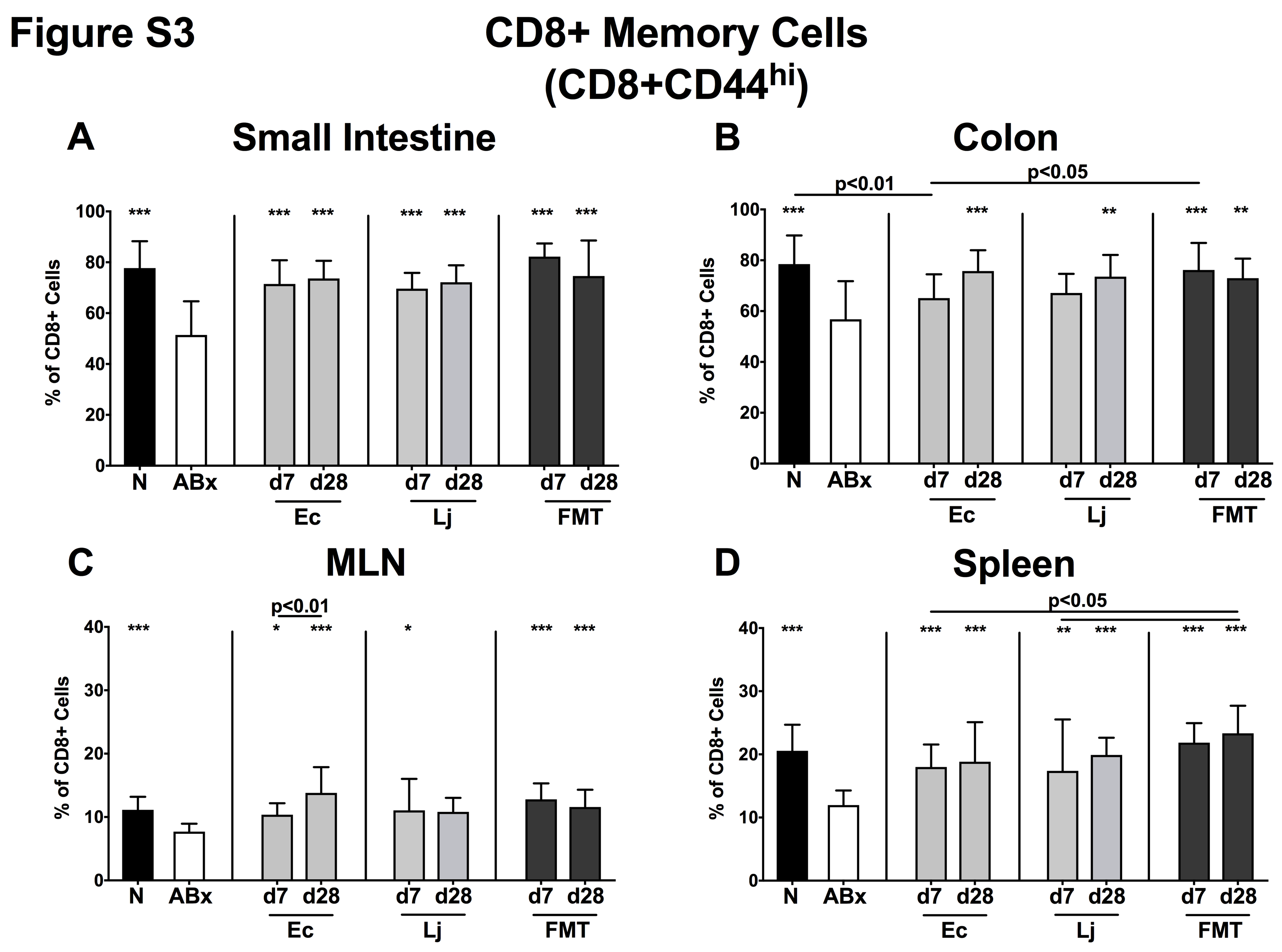

Supplement: Supplementary file 3 [file Image_3.TIFF]

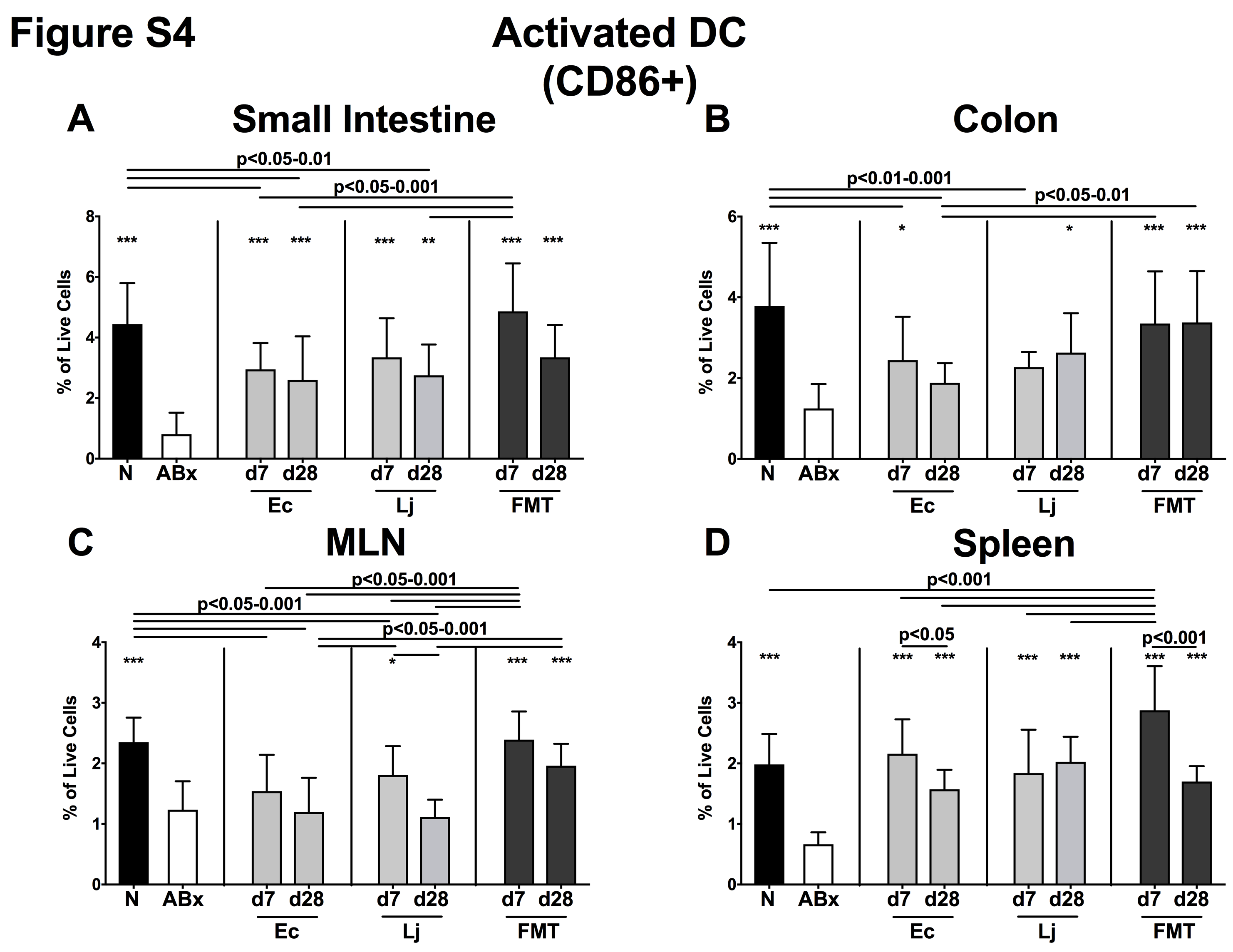

Supplement: Supplementary file 4 [file Image_4.TIFF]
